# Formulation-specific dose–response in the serum proteome of healthy dogs following cannabidiol administration

**DOI:** 10.3389/fvets.2026.1803263

**Published:** 2026-05-13

**Authors:** Wutthiwong Theerapan, Sasithorn Limsuwan, Jatuporn Rattanasrisomporn, Sekkarin Ploypetch, Natthasit Tansakul

**Affiliations:** 1 Department of Companion Animal Clinical Sciences, Faculty of Veterinary Medicine, Kasetsart University, Bangkok, Thailand; 2 Institute of Food Research and Product Development, Kasetsart University, Bangkok, Thailand; 3 Special Research Incubator Unit for Cannabis-Hemp and Phytochemicals in Veterinary Medicine, Faculty of Veterinary Medicine, Kasetsart University, Bangkok, Thailand; 4 Department of Clinical Sciences and Public Health, Faculty of Veterinary Science, Mahidol University, Nakhon Pathom, Thailand; 5 Department of Pharmacology, Faculty of Veterinary Medicine, Kasetsart University, Bangkok, Thailand

**Keywords:** cannabidiol (CBD), dog, formulation, lipid metabolism, proteomics

## Abstract

Cannabidiol (CBD) is increasingly being used in veterinary medicine; however, its systemic molecular effects in dogs remain poorly characterized. This study employed label-free quantitative proteomics to compare the serum proteomic responses of healthy dogs (*n* = 18) after 30 days of oral CBD delivery via three distinct matrices: hemp by-product feed pellets (F), CBD-infused oil (O), and semi-solid treat (SN). The verified chronic doses differed among the groups. Multivariate analysis revealed distinct formulation-specific proteomic signatures, with the F group clustered separately from the O and SN groups. Despite dose and matrix variations, all groups shared a core metabolic response characterized by downregulation of apolipoproteins (APOA4, APOC3, APOC1, and APOH) and upregulation of hemoglobin subunits (HBA and HBB), indicating CBD-mediated modulation of lipid metabolism and redox homeostasis. The high-exposure groups (O, SN) uniquely exhibited upregulation of proteins involved in vascular integrity and tissue scaffolding (e.g., TGFB1, PDGFRB, and VWF), while the SN group also showed induction of immunomodulatory and cytoprotective markers, such as clusterin (CLU). These findings demonstrate that the CBD delivery matrix critically influences systemic bioavailability and the scope of proteomic remodeling. Although all formulations engage core metabolic pathways, high-bioavailability formats induce additional signatures suggestive of vascular stabilization and stress resilience, providing a molecular rationale for optimizing CBD-based therapeutic formulations in canine medicine.

## Introduction

The integration of cannabidiol (CBD), a major non-psychoactive phytocannabinoid derived from *Cannabis sativa*, into veterinary clinical practice represents a significant paradigm shift in the management of chronic conditions in companion animals (1). In recent years, a growing number of pet owners have turned to CBD-containing products as natural, over-the-counter remedies for a variety of behavioral and physiological issues, including osteoarthritis, epilepsy, and anxiety (2). This trend is reflected in large-scale community science data, such as those from the Dog Aging Project, which indicate that approximately 7.3% of companion dogs in the United States receive CBD or hemp supplements, which is significantly higher in senior dogs and those diagnosed with chronic health conditions (3). Despite this widespread adoption, the transition of CBD from an empirical supplement to a reliable, mechanism-informed therapeutic is hampered by a persistent knowledge gap regarding its systemic molecular effects and the variability in its oral bioavailability reported in once- and twice-daily pharmacokinetic studies (2, 4, 5).

The diverse effects of CBD are generally attributed to its complex interactions within the “endocannabinoidome,” a broader signaling network that extends beyond the classical cannabinoid receptors CB1 and CB2 (2). These interactions include modulation of peroxisome proliferator-activated receptors (PPARs), transient receptor potential (TRP) channels, and serotonin receptors (6). While clinical outcome studies and safety assessments have affirmed the general tolerability of CBD in healthy dogs, these reports provide limited high-resolution insights into the systemic physiological adaptations induced by prolonged administration (7, 8). To address this deficiency, mass spectrometry-based proteomics has emerged as a powerful technology for comprehensively characterizing plasma and serum proteomes, offering a high-precision snapshot of biological flux, drug responses, and vascular integrity (9).

The application of label-free quantitative (LFQ) proteomics has proven instrumental in elucidating molecular pathways implicated in health and disease (10). Preliminary proteomic studies in rodent models and human cell lines have suggested that CBD can remodel the expression of proteins involved in inflammation, oxidative stress, and lipid metabolism (11, 12). However, the direct effects of different chronic CBD formulations on canine serum proteomes remain largely unknown. The present analysis addresses this critical gap by evaluating the molecular responses in healthy dogs following 30 days of oral CBD delivered via three distinct matrices: a hemp by-product feed pellet, CBD-infused oil, and semi-solid treat. This investigation reframed the observed differences in the verified dose and systemic exposure as a central finding, linking the physicochemical properties of the delivery matrix to the resulting proteomic remodeling.

## Materials and methods

### Experimental design and quantitative proteomics workflow

The study utilized a parallel-group design involving 18 healthy mixed-breed dogs, aged 2 to 5 years, with a mean body weight of 18.53 ± 2.95 kg. Following a minimum 25-day acclimatization period on a standard commercial diet, the subjects were randomized into three dietary groups: hemp byproduct mixed feed pellets (F), oral CBD-infused oil (O), and CBD oil-mixed semi-solid treat (SN) for a 30-day administration period. All animals were fed once daily in the morning. To minimize nutritional confounding, the three dietary formulations were designed such that total daily intake (basal diet plus any supplement) was isocaloric and isonitrogenous across groups, with similar proximate compositions (Supplementary Table 1). All experimental procedures were approved by the Institutional Animal Care and Use Committee of Kasetsart University (Protocol number: ACKU66-VET-029), and all dogs maintained a normal health status throughout the study.

### Formulation preparation and verified dose quantification

All preparations underwent analytical verification of the CBD content via HPLC. The experimental design initially aimed to standardize the targeted daily dose of CBD to approximately 24 mg per dog. However, the verified chronic doses differed. The F group (Hemp by-product Feed) received hemp by-product integrated into the feed pellet. The analysis confirmed a CBD concentration of 0.06 ± 0.005 mg/g feed, delivering an estimated 18 mg CBD per 300 g daily ration. Group O (oral CBD-fused oil) received high-purity CBD solubilized in refined oil (24 mg/mL), accurately achieving the target dose of 24 mg. The SN group (CBD oil-mixed snack) received GMP-certified treatment, which provided the highest verified dose of 28 ± 0.25 mg per treat. The O and SN groups received the same basal diet (without hemp by-product), with CBD oil or semi-solid treat administered as a separate supplement 30 min after morning feeding. The ingredient differences in Supplementary Table 1 represent necessary adjustments to balance macronutrients and energy across groups; however, these compositional differences cannot be excluded as potential contributors to the observed proteomic effects. Based on a previous study (4) reporting peak CBD absorption at 2–3 h post-administration, blood sampling was performed at 3 h after feeding.

### Sample collection and pooled proteomics design

Whole blood was collected via standard venipuncture into clot activator tubes at baseline (Day 0) and 30 days after chronic intake. Serum was separated by centrifugation and stored at −20 °C. Thirty individual serum samples were used for discovery profiling. To manage the high inter-individual biological variation common to discovery-based proteomics, a robust pooling strategy was implemented. Each experimental group consisted of five biological replicates (*n* = 5 pools), with each pool containing 100 μL of serum from six individual dogs. The expression of proteins in a pooled sample typically aligns with the average expression levels in individual samples within the pool. This approach effectively reduces biological variability and enhances the statistical power to discern treatment differences across population (13).

### Sample preparation, LC–MS/MS, and label-free quantification

Serum samples (50 μL) were mixed with 100 μL of incubation buffer (50 mM Bis-Tris Propane, pH 6.5; 150 mM NaCl) and prewashed magnetic beads. The suspensions were incubated at 37 °C for 30 min with agitation. After magnetic separation, the bead-bound material was washed with washing buffer. Proteins were denatured, reduced, and alkylated on beads using lysis buffer containing 100 mM Tris (pH 8.0), 40 mM Chloroacetamide, and 10 mM TCEP, followed by 95 °C for 10 min. Proteins were digested and the resulting peptides were eluted, acidified, and quantified.

Dried peptides were reconstituted in 30 μL 0.1% formic acid (FA). Peptide concentrations were determined using Pierce™ Colorimetric/Fluorometric Peptide Assay (Thermo Fisher Scientific). One μg of peptide per sample was injected into a nanoflow-reversed-phase LC system coupled to a Q Exactive Plus mass spectrometer. Mobile phase A contained 0.1% FA in water, and mobile phase B contained 0.1% FA in 80% acetonitrile. After pre-equilibration at 5% B, a linear gradient of 44% B was applied over 35 min. The separation was performed on an Acclaim PepMap100 C18 analytical column at 300 nL/min. The mass spectrometer was operated in data-dependent acquisition (DDA) mode, with one MS1 survey scan (m/z 300–1,650, 60,000 resolution), followed by MS/MS of the top 15 precursors (15,000 resolution). Dynamic exclusion was set at 20s. Automatic gain control (AGC) targets were 3 × 10^6^ (MS1) and 1 × 10^5^ (MS2).

### Bioinformatics and statistical analysis

Raw files were processed using the FragPipe workflow (MSFragger/Philosopher/IonQuant) and searched against the *Canis lupus familiaris* reference proteome (UP000002254). Label-free quantification (LFQ) included match-between-run. Proteins were retained for analysis only if they were quantified in ≥3 of the 5 replicates within at least one group. Missing values were imputed from a normal distribution (width = 0.3, downshift = 1.8). Statistical analysis was performed using Student’s t-test in Perseus with Benjamini–Hochberg correction. Proteins were classified as significantly differentially expressed (DEP) at a threshold of |log2 fold-change| ≥ 1 (≥2-fold change) and ≤ 0.05.

Univariate and multivariate analyses were performed using MetaboAnalyst 6.0 to identify protein expression shifts induced by CBD administration^1^ (doi:10.1038/s41596-023-00950-4). For pairwise comparisons, significant differences were determined using a two-sample t-test, with proteins deemed significantly altered if they met a False Discovery Rate (FDR) threshold of *p* < 0.05. To evaluate proteomic variations across the three experimental groups following 30 days of oral CBD administration, we employed multivariate analysis including Partial Least Squares Discriminant Analysis (PLS-DA) and one-way ANOVA. For ANOVA, post-hoc comparisons were conducted using Fisher’s Least Significant Difference (LSD), requiring both a raw *p* < 0.05, and an FDR-adjusted *p* < 0.05. The candidate proteins were further analyzed using the STITCH 5.0 database (accessed January 9, 2025) to elucidate their functional roles and to map protein–protein interactions specifically associated with tetrahydrocannabinolic acid (THCA) synthase and cannabidiolic acid (CBDA) synthase (14). Functional annotation was performed based on Gene Ontology (GO) categories, including biological processes, cellular components, and molecular functions. Additionally, enrichment analysis of differentially expressed proteins (DEPs) was conducted using ShinyGO v0.85, to identify significantly overrepresented GO terms, and Kyoto Encyclopedia of Genes and Genomes (KEGG) pathways, applying a statistical significance cutoff of FDR < 0.05 (accessed on January 13, 2025) (doi: 10.1093/bioinformatics/btz931).

## Results

### Protein identification and profile separation

**Figure 1:**
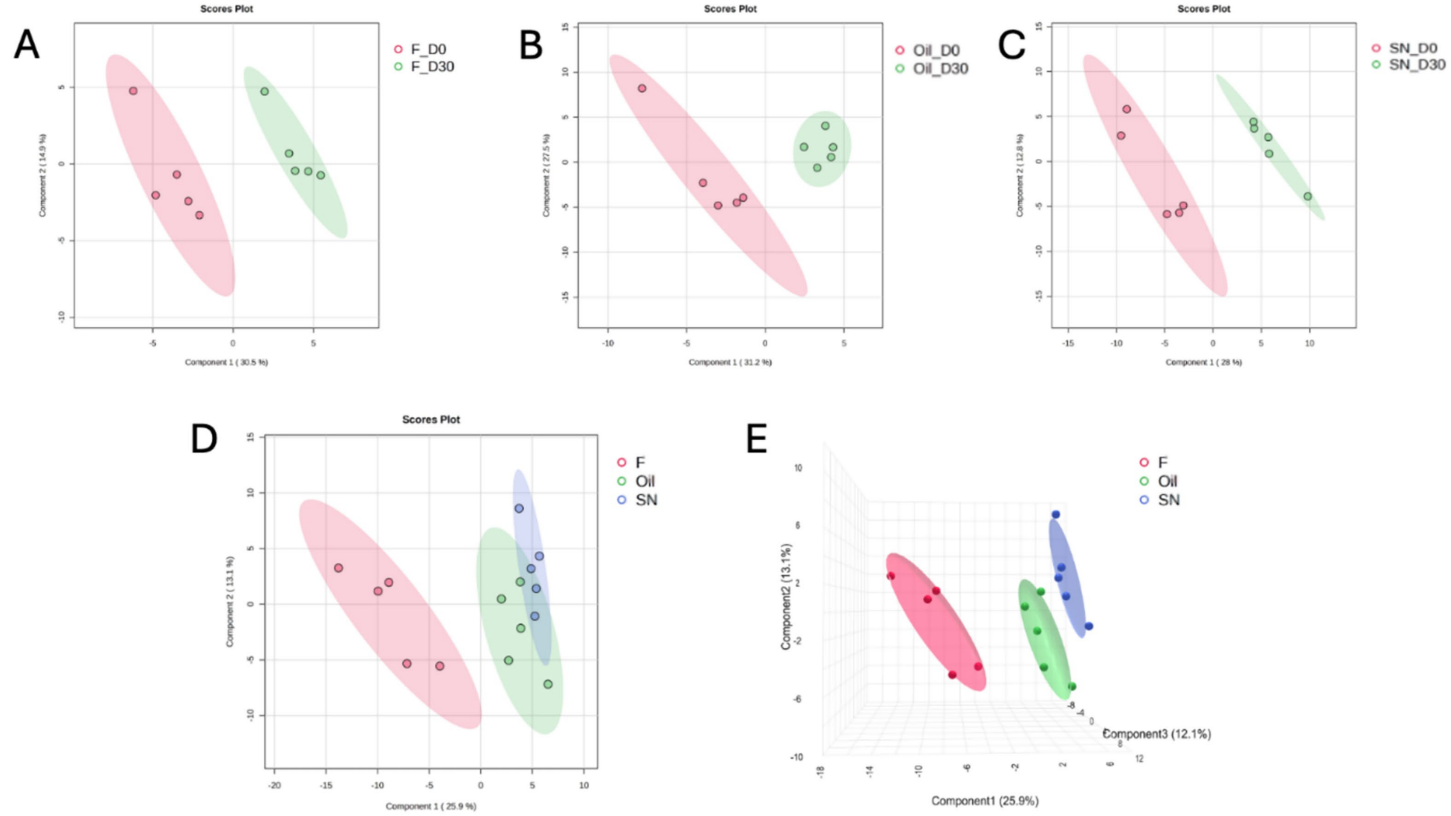
Partial least squares-discriminant analysis (PLS-DA) score plots illustrating proteomic profile shifts across treatment groups. Two-dimensional (2D) and three-dimensional (3D) PLS-DA score plots demonstrate the discrimination between experimental groups. Pairwise comparisons between Day 0 (red) and Day 30 (green) for groups F, Oil, and SN, respectively, showing significant temporal separation along Component 1 **(A–C)**. Multigroup 2D comparisons showing the spatial distribution of F (red), Oil (green), and SN (blue) groups at Day 30 **(D)**. 3D score plot providing an integrated view of the separation between all three treatment groups **(E)**.

Across the three administration groups, the number of quantified proteins was 86 in SN, 63 in F, and 64 in O. Within-group comparisons (Day 0 versus Day 30) demonstrated separation in the 2D PLS-DA plot, confirming temporal changes induced by all three CBD-containing products (Figures 1A–C; Supplementary Table 2).

By Day 30, supervised multivariate analysis using Partial Least Squares Discriminant Analysis (PLS-DA) revealed distinct proteomic signatures across the experimental cohorts. Both the 2D and 3D score plots demonstrated a robust separation of the F group from the O and SN groups, predominantly along component 1 (explaining 25.9% of the total variance). This clear spatial segregation suggests that the molecular profile of the F group was fundamentally distinct from that of the other treatments (Figures 1D,E). Conversely, the O and SN groups exhibited significant proximity and partial overlap within the 95% confidence ellipsoids, particularly in the 3D projection. This clustering pattern indicates that the administration of directed oil (O) and the matrix combination (SN) elicited a convergent proteomic response, suggesting that these two treatments drive the dog’s response toward shared molecular information that is qualitatively different from that of the F group.

### Intragroup differential protein expression (Day 0 vs. Day 30)

Proteomic profiling of samples at Day 30 compared to baseline (Day 0) revealed distinct group-specific molecular shifts (Supplementary Table 3). In the F group, four proteins, glyceraldehyde-3-phosphate dehydrogenase (GAPDH), hemoglobin subunit alpha (HBA), hemoglobin subunit beta (HBB), and pleckstrin (PLEK), were significantly upregulated, whereas apolipoprotein A-IV (APOA4) and apolipoprotein C-III (APOC3) were downregulated. In the O (Oil) group, increased expression was observed for deoxyribonuclease-1 (DNASE-1), HBA, and HBB, whereas apolipoprotein C-I (APOC1), beta-2-glycoprotein I, and apolipoprotein H (APOH) were significantly downregulated. Notably, the SN group exhibited the most extensive proteomic modulation, with seven proteins significantly upregulated: acidic leucine-rich nuclear phosphoprotein 32 family member A (ANP32A), clusterin (CLU), glyceraldehyde-3-phosphate dehydrogenase (GAPDH), hemoglobin subunit alpha (HBA), hemoglobin subunit beta (HBB), pleckstrin (PLEK), and von Willebrand factor (VWF). Conversely, the expression of hepatocyte growth factor activator (HGFAC) and metalloproteinase inhibitor 1 (TIMP1) was significantly suppressed in the SN cohort (Supplementary Table 3).

### Intergroup differential protein expression (F vs. O vs. SN groups at Day 30)

A comparative analysis among the groups at Day 30 was conducted to elucidate the molecular signatures associated with systemic CBD exposure. This multi-group comparison identified 11 proteins that were significantly upregulated in the O and SN groups (Supplementary Table 3). Notably, the upregulated proteome was enriched with key regulators of vascular and structural integrity, including desmin (DES), endoplasmin (HSP90B1), fibronectin (fragment) (FN1), Ig kappa chain V region GOM, Ig mu chain C region, platelet-derived growth factor receptor beta (PDGFRB), transferrin receptor protein 1 (TFRC), transforming growth factor beta-1 proprotein (TGFB1), tight junction protein ZO-2 (TJP2), vascular cell adhesion protein 1 (VCAM1), and von Willebrand factor (VWF) (Supplementary Table 3).

### Functional annotation and KEGG pathway enrichment analysis

**Figure 2:**
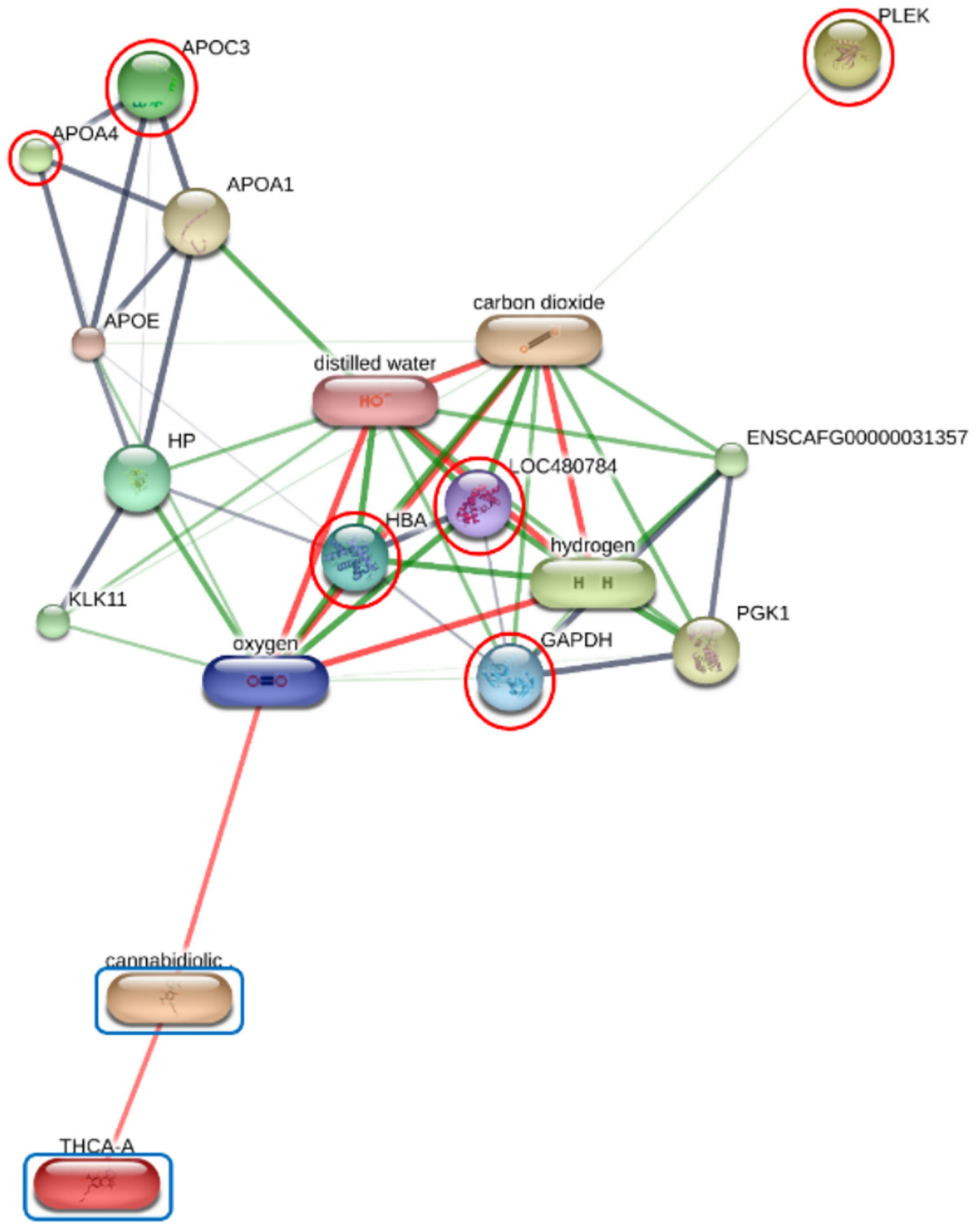
Protein-chemical interaction network of prioritized differentially expressed proteins (DEPs) and cannabinoids in the F group. The network illustrates the regulatory relationships in the F group between six prioritized DEPs including GAPDH, HBA, HBB (LOC480784), PLEK, APOA4, and APOC3 (marked with red circles) and the cannabinoids cannabidiolic acid (CBDA) and tetrahydrocannabinolic acid (THCA) (blue boxes). GAPDH, HBA, HBB (LOC480784), PLEK, APOA4, and APOC3 are integrated into a network with metabolic regulators and gas molecules, highlighting the baseline interaction landscape influenced by cannabinoid presence. Nodes represent proteins or chemical compounds, while edges represent predicted or known interactions. Edge thickness and color (green and red) indicate the type and strength of the supporting evidence for each interaction.

**Figure 3:**
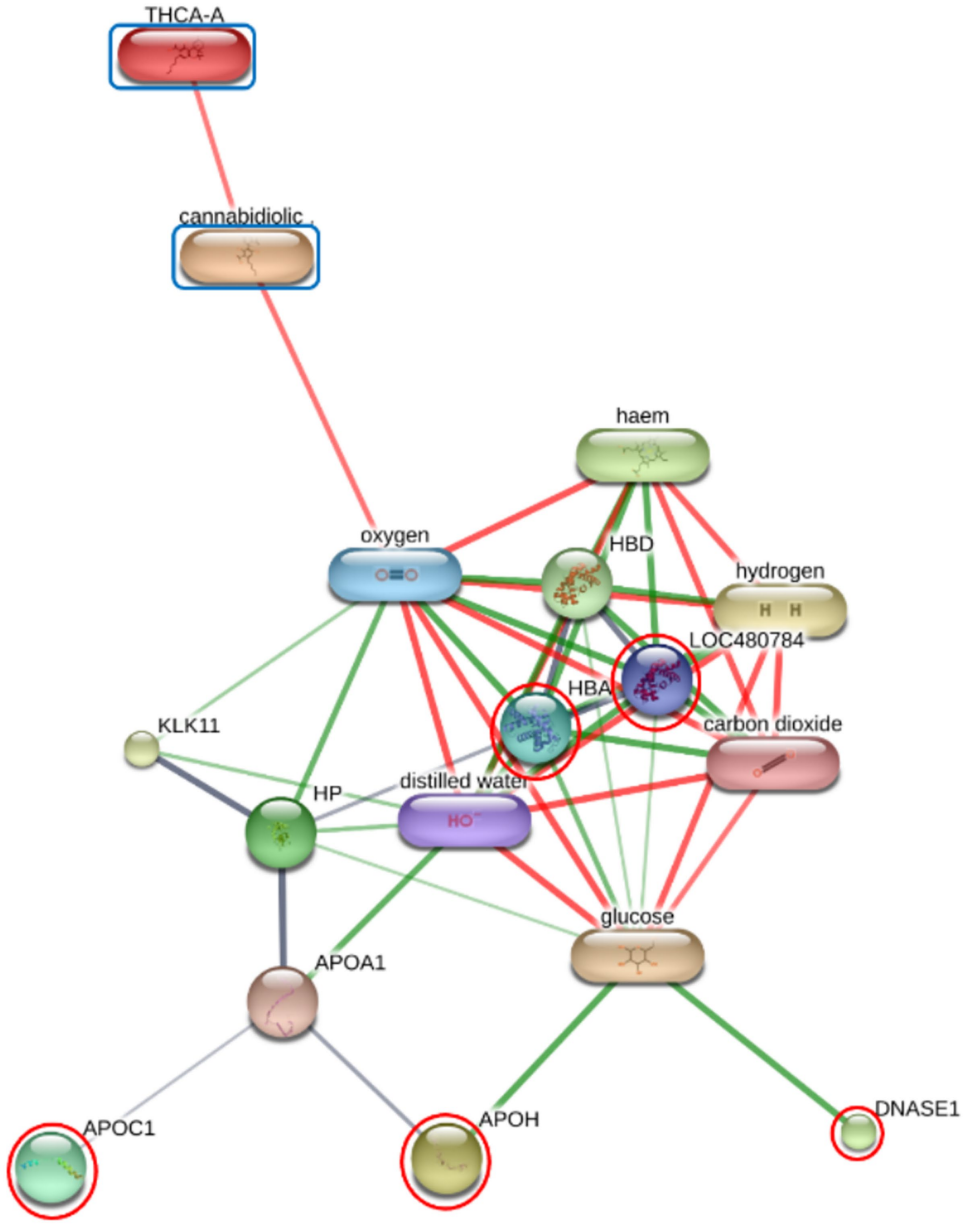
Protein-chemical interaction network of prioritized differentially expressed proteins (DEPs) in the O group. The network represents the modeled regulatory relationships between five prioritized differentially expressed proteins (DEPs; highlighted with red circles) and the cannabinoids cannabidiolic acid (CBDA) and tetrahydrocannabinolic acid (THCA) (blue boxes). *n* this group, DNASE1, HBA, HBB (LOC480784), APOC1, and APOH are integrated into a metabolic scaffold centered around oxygen and glucose signaling. Nodes represent proteins or chemical compounds, while edges represent predicted or known interactions. Edge thickness and color (green and red) indicate the type and strength of the supporting evidence for each interaction.

**Figure 4:**
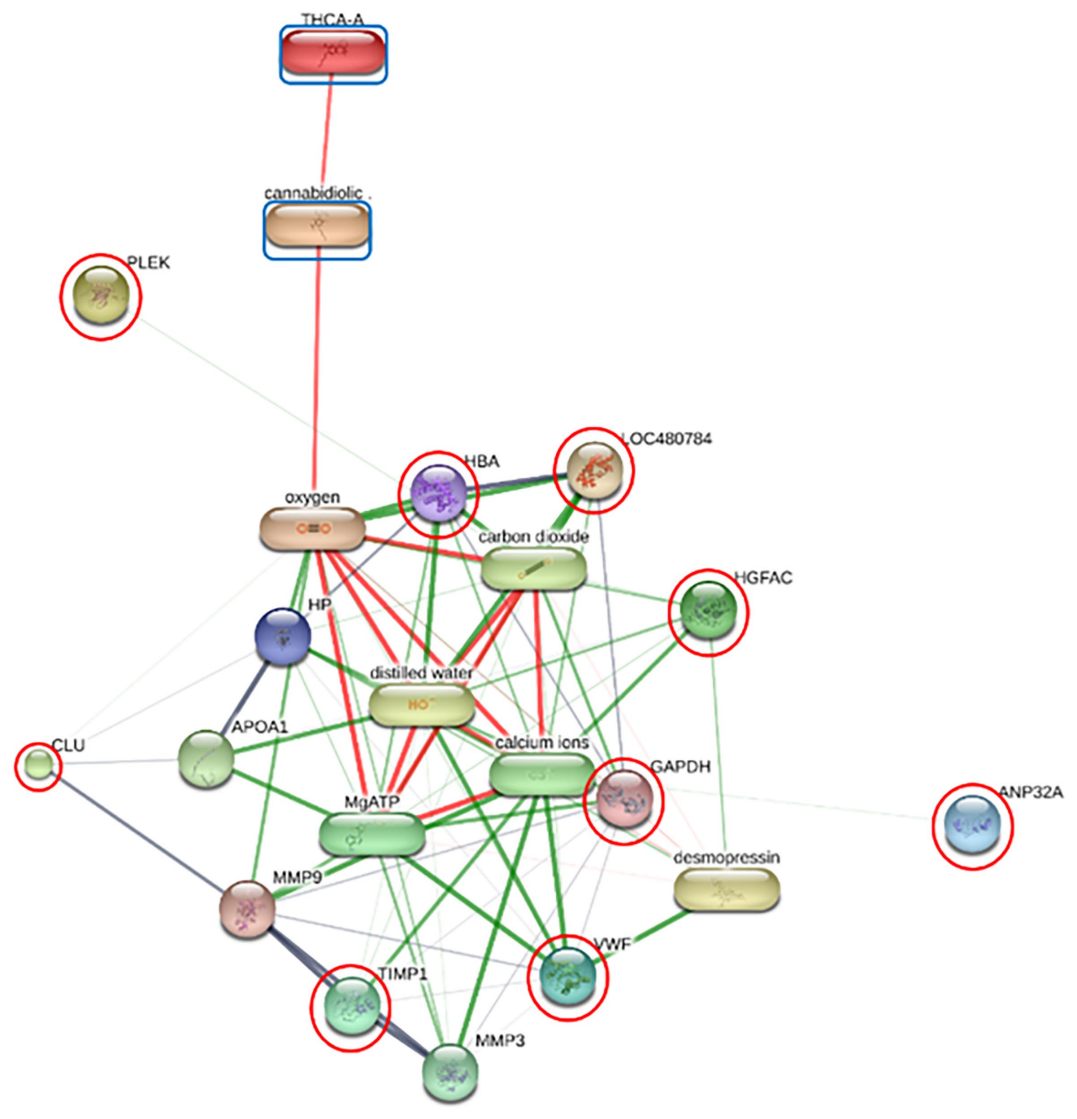
Protein-chemical interaction network of prioritized differentially expressed proteins DEPs in the SN group. The network illustrates the modeled regulatory relationships between nine prioritized differentially expressed proteins (DEPs; highlighted with red circles) and the cannabinoids cannabidiolic acid (CBDA) and tetrahydrocannabinolic acid (THCA) (blue boxes). In the SN group, ANP32A, CLU (Clusterin), GAPDH, HBA, HBB (LOC480784), PLEK, VWF (von Willebrand factor), HGFAC, and TIMP1 are integrated into a complex regulatory web. Nodes represent proteins or chemical compounds, while edges represent predicted or known interactions. Edge thickness and color (green and red) indicate the type and strength of the supporting evidence for each interaction.

Functional enrichment of the differentially expressed proteins (DEPs) was performed using ShinyGO v 0.85 to identify significantly overrepresented Gene Ontology (GO) terms and KEGG pathways. In the F group, the regulatory relationships between six prioritized DEPs—including GAPDH, HBA, HBB, PLEK, APOA4, and APOC3—and the cannabinoids CBDA and THCA were modeled to establish a baseline interaction network (Figure 2; Supplementary Table 4). Similar interaction analyses were conducted for the O group (comprising DNASE1, HBA, HBB, APOC1, and APOH; Figure 3) and SN group (ANP32A, CLU, GAPDH, HBA, HBB, PLEK, VWF, HGFAC, and TIMP1; Figure 4).

**Figure 5:**
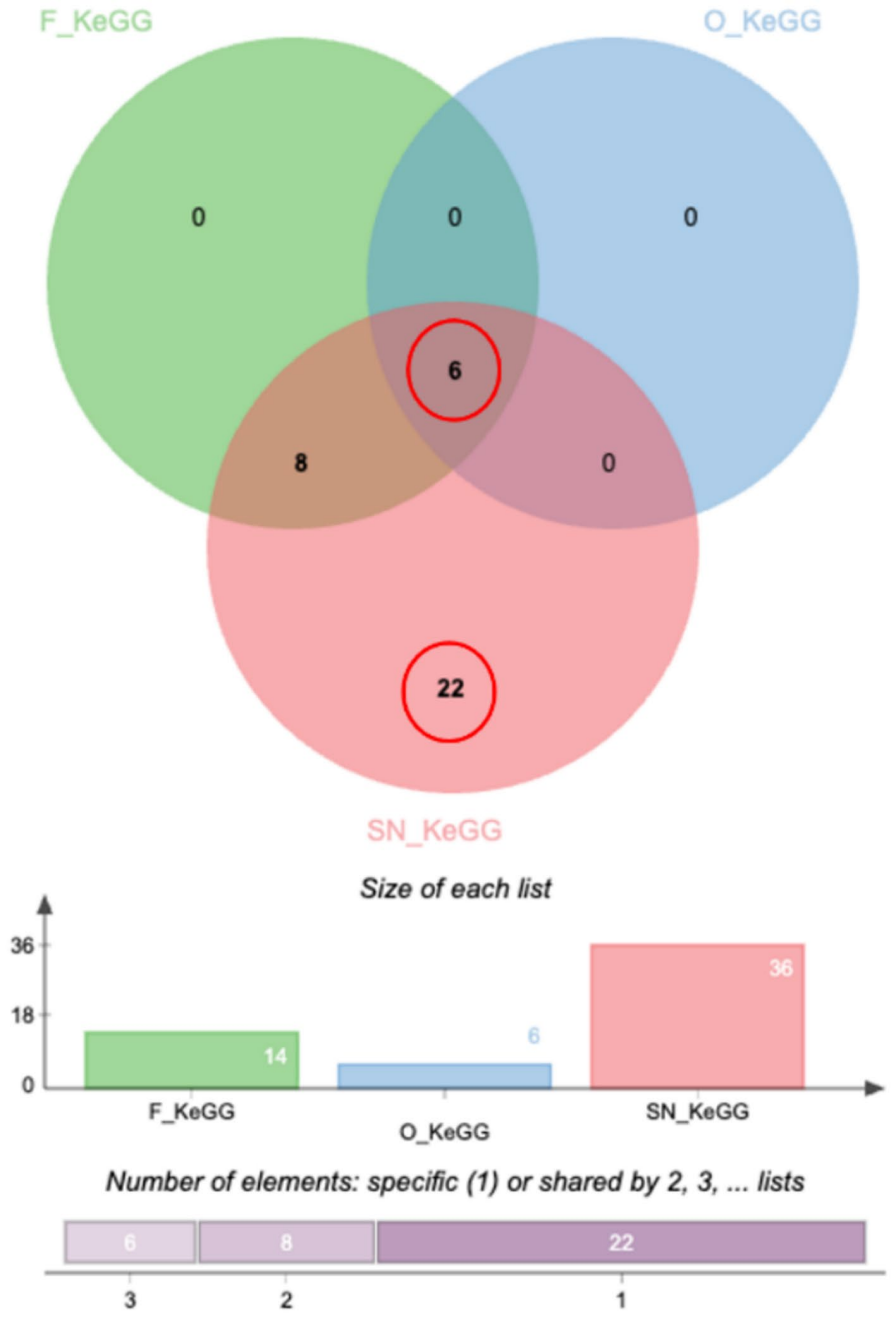
Comparative KEGG pathway enrichment analysis across treatment cohorts. A Venn diagram illustrates the overlap of enriched KEGG pathways among the F (green), O (blue), and SN (red) cohorts following 30 days of CBD administration. A core metabolic axis, represented by the central intersection of all three groups (*n* = 6 pathways), was identified as a shared biological response. The associated bar chart (“Size of each list”) details the total number of enriched pathways per group (F: 14, O: 6, SN: 36), while the bottom horizontal bar indicates the distribution of pathways that are specific to a single cohort (*n* = 22 for SN) or shared between two (*n* = 8) or all three (*n* = 6) groups.

Comparative KEGG pathway enrichment across all cohorts, visualized via a Venn diagram, identified a core metabolic axis common to all groups after 30 days of CBD administration. This shared response was characterized by significant modulation of cholesterol metabolism, vitamin and fat digestion/absorption, PPAR signaling pathway, and lipid-associated atherosclerosis pathways (Figure 5; Supplementary Table 5). Notably, the SN formulation, which achieved the highest sustained systemic exposure, uniquely activated robust metabolic and hemostatic compensatory mechanisms, while concurrently suppressing pro-inflammatory and cellular growth signaling modules.

## Discussion

This study employed a parallel design using three distinct CBD delivery matrices, hemp by-product mixed feed pellets (F), oral CBD-infused oil (O), and a CBD oil-mixed semi-solid treat (SN), to directly compare their effects on the serum proteome. Quantitative label-free proteomics provides an unbiased, high-resolution view of the systemic physiological adaptations induced by these interventions. The robust temporal changes observed across all groups affirmed the utility of serum proteomics as a sensitive indicator of the systemic nutritional response. Notably, the multivariate analysis revealed that the F group formed a unique proteomic cluster that was distinctly separated from the O and SN groups. This divergence suggests that the proteomic shifts were shaped by a combination of dose-dependent and matrix-driven effects, with the most extensive modulation occurring in the SN group, followed by the O and F. Despite these formulation-specific gradients, a core set of differentially expressed proteins and enriched pathways consistently points to a fundamental role for CBD in regulating lipid homeostasis and energy metabolism, irrespective of the delivery vehicle.

### Multivariate characterization of formulation-specific proteomic remodeling

A primary finding that elucidates this relationship is the significant role of the delivery matrix in determining the systemic CBD exposure. This disparity (SN > O > F) provided an essential context for the observed molecular outcomes. The reduced bioavailability in the F group is likely a consequence of the mixing and pelletization process, where thermal and mechanical stresses during integration into the basal feed may potentially reduce CBD content or degrade cannabinoids. However, as pre- and post-processing CBD concentrations were not quantified in the present study, this interpretation remains hypothetical and warrants further investigation. Furthermore, the sequestration of CBD within the fibrous matrix of the hemp by-product may limit its intestinal liberation, a phenomenon observed in other pharmacokinetic studies where oral bioavailability in dogs is typically low, often ranging from 13 to 19% (15).

Conversely, the superior proteomic remodeling and higher verified dose observed in the SN group suggested that the semi-solid matrix functioned as a protective vehicle that may have enhanced absorption or minimized pre-ingestion degradation. This interpretation is consistent with pharmacokinetic evidence indicating that oral CBD exposure in dogs is significantly influenced by co-administered lipids (7). Administration via lipid-rich vehicles, such as oils or treats, has been shown to substantially increase maximum plasma concentration and area under the curve (5, 7). Kamutchat et al. (7) reported serum CBD levels, dosing once daily, ranging from 21 to 244 ng/mL in the oil group (5 mg/kg) compared to 3 to 43 ng/mL in the snack group (50 mg/dog), confirming that formulation and dosing fundamentally influence systemic exposure. These pharmacokinetic data align with our finding of formulation-dependent proteomic remodeling, suggesting that the SN and O formulations, by utilizing lipid-rich vehicles, likely overcame the absorption limitations of the feed pellet, resulting in their more pronounced molecular shifts. Overall, differences in proteomic signatures should be viewed as a combined effect of the delivery matrix and the resulting systemic dose.

Supervised multivariate analysis via Partial Least Squares Discriminant Analysis (PLS-DA) was used to visualize the systemic molecular changes induced by chronic CBD intake. PLS-DA revealed robust temporal separation in all groups by Day 30, confirming that CBD intake measurably remodeled the serum proteome. The magnitude of these proteomic shifts correlated with both the administered dose and the bioavailability of the delivery matrix. At the study endpoint, distinct clustering was evident between the treatment groups. The F group was clearly separated from the O and SN groups. This indicated a fundamentally different molecular state at lower exposure levels. In contrast, the O and SN groups, which received higher bioavailable doses, clustered closely, with overlapping confidence intervals. This convergence implies that once a sufficient CBD exposure threshold is reached, the physiological response of the host aligns with a common high-dose molecular signature.

### Metabolic adaptations and the core response to CBD

Despite formulation-specific differences, a foundational molecular signature common to all three experimental groups was the significant modulation of the proteins involved in lipid transport and energy metabolism. This shared proteomic signature is characterized by a marked upregulation of hemoglobin subunits (HBA and HBB), suggesting a potential systemic adjustment in oxygen transport or redox homeostasis. Concurrently, we observed a coordinated downregulation of key apolipoproteins, including APOA4, APOC3, APOC1, and APOH, indicating systemic recalibration of lipid metabolism and transport pathways (16, 17). This shared response suggests that CBD engages a central metabolic axis in the canine host independent of the delivery matrix.

Further supporting this functional convergence, enrichment analysis identified common perturbations in core metabolic processes. Notably, the most profoundly affected proteins, particularly apolipoproteins, are among the most abundant and functionally important proteins in healthy canine plasma, as recently characterized by Doulidis et al. (9). The significant downregulation of these essential plasma components demonstrates that chronic CBD administration acts as a substantial metabolic perturbation with the potential for wide-ranging systemic effects including lipid metabolism and transport (16, 18).

The nature and extent of this metabolic recalibration displayed a clear relationship with the exposure level. In the low-exposure F group, the PPI network was centered on the suppression of APOA4 and APOC3, which are functionally linked to an oxygen metabolic axis. This suggests an initial metabolic stabilization, as the repression of APOC3, a known inhibitor of lipoprotein lipase (LPL), facilitates enhanced triglyceride clearance (18, 19). The observation that even the lowest verified dose induced this change indicates that CBD may optimize lipid processing at relatively low exposure levels. This contrasts with findings in heavy marijuana users, where high THC levels have been associated with increased serum APOC3 levels, likely through the activation of hepatic CB1 receptors that promote lipogenesis (20).

The suppression of APOC1 in the Oil (O) group further supports this metabolic shift. APOC1 is the only known endogenous inhibitor of cholesteryl ester transfer protein (CETP) that mediates the exchange of cholesteryl esters and triglycerides between high-density lipoproteins (HDL) and very-low-density lipoproteins (VLDL) (16). The reduction in APOC1 may facilitate more efficient sterol transport and HDL maturation. Concurrently, downregulation of APOH (beta-2-glycoprotein I) suggests a transition toward a more stable systemic state, as APOH is often elevated in response to inflammatory or metabolic stress (21). Downregulation of APOC1 and APOH is directly linked to lipoprotein metabolism (17). This study is consistent with recent evidence that short-term CBD affects lymphatic lipid composition, and that apolipoprotein secretion underscores its direct role in modulating lipid transport (22).

The significant enrichment of pathways related to cholesterol metabolism, PPAR signaling, and lipid digestion/absorption indicates that this systemic metabolic reprogramming is likely mediated through the expanded signaling network of the “endocannabinoidome” where peroxisome proliferator-activated receptors (PPARs) serve as key molecular targets (6, 23). CBD functions as a direct PPARγ agonist and can indirectly enhance PPARs activity (6, 24). PPARα is a master regulator of lipid metabolism, and PPARγ activation contributes to potent anti-inflammatory effects (25, 26). The observed systemic downregulation of apolipoproteins is consistent with a “transrepression” mechanism, in which activated PPARs interfere with other transcription factors to suppress the expression of target genes (27). Such downregulation aligns with known physiological responses to metabolic stress, and is consistent with research showing that CBD treatment alters metabolite and transcript levels in a dose-dependent manner (12, 21). Collectively, these mechanisms may reveal how chronic CBD consumption engages central metabolic regulatory systems to induce a state of optimized lipid homeostasis and reduced inflammatory tone (6, 23, 28).

This metabolic reprogramming was further evidenced by the upregulation of glyceraldehyde-3-phosphate dehydrogenase (GAPDH) in both the F and SN groups. Traditionally viewed as a glycolytic enzyme, GAPDH is a pleiotropic protein involved in various cellular processes, including iron metabolism and response to oxidative stress (29). Its elevation may coordinate adjustments in glucose utilization and cellular energy flux in response to chronic CBD exposure.

An unexpected but consistent finding across all three formulations was the significant upregulation of hemoglobin subunit alpha (HBA) and hemoglobin subunit beta (HBB). This coordinated increase in the structural components of hemoglobin suggests a potential systemic adjustment of oxygen transport or redox homeostasis. The increase in HBA and HBB at Day 30 compared to Day 0 indicates an adaptation that may enhance systemic resilience to metabolic perturbations (30). Similar observations have been made in rodent models, where *C. sativa* leaf powder supplementation ameliorated hemoglobin levels and accelerated functional recovery after nerve injury (31). In the context of the present study, this oxygen metabolic axis likely served as a compensatory mechanism to support the metabolic demands of the recalibrated lipid transport system. The interaction networks modeled for each group consistently positioned HBA and HBB as central nodes functionally linked to both energy flux (GAPDH) and platelet activation (PLEK).

### Vascular stabilization, tissue scaffolding and hemostasis

Intergroup comparison conducted on Day 30 revealed a distinct molecular signature unique to the high-exposure cohorts (O and SN). This signature is characterized by the upregulation of a complex of proteins involved in maintaining vascular integrity and structural resilience, specifically the TGFB1/PDGFRB/VWF axis.

Specific upregulation of transforming growth factor beta-1 (TGFB1) and platelet-derived growth factor receptor beta (PDGFRB) are critical indicators of systemic vascular adaptation. This ligand-receptor pair is essential for the recruitment of pericytes to the mature vasculature and for maintaining endothelial quiescence (32, 33). The activation of PDGFRB observed in the present study may indicate its potential to enhance biological processes involved in musculoskeletal repair. This includes the proliferation of tendon cells and synthesis of collagen following treatment with PDGF-BB in a canine model, as previously reported (34, 35).

The presence of this signature in the high exposure groups suggests that reaching a specific CBD concentration threshold triggers a pro-healing microenvironment. TGFB1 is a master regulator of tissue homeostasis with context-dependent roles (36, 37), often mediating antiproliferative stability and repair in models of tissue remodeling and osteoarthritis (38, 39). The observed upregulation may reflect the CBD’s potential to support the structural scaffold of the circulatory and musculoskeletal systems, providing a molecular rationale for its use in managing conditions such as osteoarthritis and soft tissue injuries.

In the SN group, von Willebrand factor (VWF) emerged as a central protein within the interaction network. VWF is a large multimeric glycoprotein critical for platelet adhesion and the stabilization of factor VIII. While elevated VWF is typically considered a marker of endothelial activation or injury in inflammatory diseases (40, 41). Its coordinated increase with other vascular stabilizers such as PDGFRB and tight junction protein ZO-2 (TJP2) points toward a compensatory enhancement of hemostatic readiness and vascular endothelial integrity (42).

This response is potentially modulated by CBD’s known effects on vascular tone and endothelial function through the endocannabinoid system (43, 44). The translational relevance of this finding is significant for conditions marked by vascular dysfunction or fragility, suggesting that high-bioavailability CBD formulations may offer systemic benefits to circulatory health.

### Immunomodulation and stress resilience

The SN formulation, which achieved the highest verified CBD dose, uniquely recruited signaling modules associated with immunomodulation and the adaptive cellular stress response. This group clarified the activation of pathways related to IL-17 and TNF signaling, which play roles in hemostasis and inflammation. The recruitment of these master inflammatory regulators at the highest dose suggests that CBD acts as a system-level immunomodulator rather than a simple immunosuppressant (45, 46). This aligns with preclinical evidence that cannabinoids can exert biphasic or context-dependent effects on cytokine production, fine-tuning the balance between pro- and anti-inflammatory states (46–48). In canines, this immunomodulatory potential has been demonstrated in peripheral blood mononuclear cells (PBMCs), where CBD decreased the production of pro-inflammatory cytokines such as TNF-α, IL-1β, and IL-6 in a dose-dependent manner (1, 48).

The SN group uniquely exhibited the induction of Clusterin (CLU), an ATP-independent extracellular molecular chaperone. CLU plays a vital role in protecting against endoplasmic reticulum (ER) stress, inhibiting the aggregation of misfolded proteins, and promoting cell survival (49). Considering that CLU expression is frequently reduced in both neurodegenerative diseases and systemic inflammatory states, the enhancement of CLU expression through high-dose CBD administration may play a crucial role in providing cytoprotective and neuroprotective effects (50).

The observed induction of CLU and modulation of nuclear phosphoproteins such as ANP32A provide a molecular basis for these clinical reports, suggesting that high-bioavailability CBD supports the host’s ability to manage proteostatic and cellular stress (51, 52). In addition, this mechanism may underlie the behavioral changes following CBD intervention. Data from the Dog Aging Project indicate that dogs administered CBD products for multiple years show a significant decline in the intensity of aggressive behaviors over time (3).

Although the proteomic signatures identified in this study provide novel insights, it is essential to interpret them within the context of established pharmacokinetic variability in canine species. Recent clinical reports, such as the study by Lyons et al. (53), have noted that plasma cannabinoid concentrations can be highly variable across individual dogs and may not always correlate directly with clinical outcomes such as analgesia following surgery.

In the present study, physiological interpretations must be viewed as potential associations rather than definitive causalities. For example, while the upregulation of VWF and PDGFRB suggests vascular stabilization, the biological outcome depends on the preexisting state of the animal. In healthy dogs, these changes may represent a strengthening of the physiological scaffold, whereas in diseased models, they may reflect different stages of tissue repair or compensation (9).

Furthermore, technical limitations, such as the relatively low number of quantified proteins and the sub-chronic 30-day administration period, mean that these results may not fully encompass the molecular adaptations occurring over long-term use. The absence of a true negative control means that environmental or temporal effects cannot be completely excluded. However, this limitation is partially mitigated by within-group comparisons (Day 0 vs. Day 30), which establish an individual baseline for each animal. In addition, diet reformulation could contribute to the observed proteomic differences. The pooled sample design, which is statistically powerful for population-level signatures, inherently masks individual-level variations in drug metabolism and responses (13). In addition, concurrent serum CBD quantification in future studies would allow direct confirmation of the exposure-response relationship. Therefore, these findings should be viewed as foundational molecular frameworks that require further validation in larger, unpooled clinical cohorts across different disease states.

## Conclusion

The formulation matrix critically determines systemic bioavailability and, thus, the scope of proteomic remodeling induced by chronic CBD in dogs. While all formulations modulate core lipid metabolism pathways, high-bioavailability formats (oil and snack) uniquely induce signatures suggestive of vascular stabilization and immunomodulation, providing a molecular basis for optimizing therapeutic formulations.

## Data Availability

The datasets presented in this study can be found in online repositories. The names of the repository/repositories and accession number(s) can be found in the article/Supplementary material.

## Footnotes

1
http://www.metaboanalyst.ca
