# Correction: Formulation-specific dose–response in the serum proteome of healthy dogs following cannabidiol administration

**DOI:** 10.3389/fvets.2026.1882983

**Published:** 2026-06-30

**Authors:** Wutthiwong Theerapan, Sasithorn Limsuwan, Jatuporn Rattanasrisomporn, Sekkarin Ploypetch, Natthasit Tansakul

**Affiliations:** 1 Department of Companion Animal Clinical Sciences, Faculty of Veterinary Medicine, Kasetsart University, Bangkok, Thailand; 2 Institute of Food Research and Product Development, Kasetsart University, Bangkok, Thailand; 3 Special Research Incubator Unit for Cannabis-Hemp and Phytochemicals in Veterinary Medicine, Faculty of Veterinary Medicine, Kasetsart University, Bangkok, Thailand; 4 Department of Clinical Sciences and Public Health, Faculty of Veterinary Science, Mahidol University, Nakhon Pathom, Thailand; 5 Department of Pharmacology, Faculty of Veterinary Medicine, Kasetsart University, Bangkok, Thailand

**Keywords:** cannabidiol (CBD), dog, formulation, lipid metabolism, proteomics

Author Sekkarin Ploypetch was erroneously assigned as corresponding author. The only corresponding author is Natthasit Tansakul.

The original version of this article has been updated.

